# Associations between occupational therapy students’ academic performance and their study approaches and perceptions of the learning environment

**DOI:** 10.1186/s12909-021-02940-0

**Published:** 2021-09-18

**Authors:** T. Bonsaksen, T. A. Magne, L. Stigen, A. Gramstad, L. Åsli, G. Mørk, S. G. Johnson, T. Carstensen

**Affiliations:** 1 Department of Health and Nursing Sciences, Faculty of Social and Health Sciences, Inland Norway University of Applied Science, Elverum, Norway; 2 Faculty of Health Studies, VID Specialized University, Sandnes, Norway; 3 Department of Occupational Therapy, Prosthetics and Orthotics, Faculty of Health Sciences, Oslo Metropolitan University, Oslo, Norway; 4 Department of Neuromedicine and Movement Science, Faculty of Medicine and Health Science, Norwegian University of Science and Technology, Trondheim, Norway; 5 Department of Health Sciences, Faculty of Medicine and Health Science, Norwegian University of Science and Technology, Gjøvik, Norway; 6 UiT The Arctic University of Norway, Tromsø, Norway; 7 Centre for care research, Tromsø, Norway; 8 Department of Occupational Therapy, Faculty of health and function, Western Norway University of Applied Sciences, Bergen, Norway

**Keywords:** Academic performance, Approaches to studying, Grade point average, Higher education, Learning environment, Occupational therapy

## Abstract

**Background:**

Relationships between students’ academic performance and their employed study approaches have been studied extensively. However, research using study approaches and learning environment factors as concurrent predictors of academic performance is sparse. There is a need to disentangle the potentially interrelated influences of individual and contextual factors on students’ academic performance.

**Objective:**

This study aimed to increase the understanding of the associations between occupational therapy students’ academic performance, and their approaches to studying, perceptions of the learning environment, and sociodemographic characteristics.

**Method:**

A cross-sectional study was designed, and 174 first-year students completed the Approaches and Study Skills Inventory for Students and the Course Experience Questionnaire, in addition to background information. Data on grades were collected from the data registries of each education institution, and associations were analyzed by multiple linear regression.

**Results:**

None of the learning environment scales were associated with grades. Adjusting for all variables, better exam results were associated with being female (*β* = 0.22, *p* < 0.01) and having higher scores on strategic approach (*β* = 0.31, *p* < 0.001) and lower scores on surface approach (*β* = -0.20, *p* < 0.01).

**Conclusion:**

The study suggests that students with a desire for obtaining good grades ought to use strategic study behaviors and avoid using surface approach behaviors. While it is important to ensure good quality of the learning environment for a variety of reasons, the learning environment did not contribute significantly to explain the students’ academic performance.

## Introduction

Exams are summative assessments traditionally used to evaluate students’ academic performance, and exam grades purport to measure students’ learning outcomes. Previous research has described factors influencing academic performance as relating to two different sources. One focuses on the student’s individual characteristics, of which some are fixed (such as gender) and others are changeable (such as motivation and study behaviors). The second focuses primarily on factors comprising the learning environment, for example the classroom climate and relationships among students [1–3]. In this article, study behaviors and learning environment factors are examined together as possible covariates to first-year occupational therapy students’ academic performance.

In relation to individual characteristics, Bonsaksen and co-workers [4] described a range of socioeconomic factors characterizing Norwegian occupational therapy students. Some sociodemographic factors – in particular higher age and female gender – have been found to contribute to determine their academic performance [5, 6]. In recent studies of occupational therapy students, having the current study program listed as the prioritized line of education at the time of enrolment has been shown to be associated with study behaviors [7, 8]. Moreover, having prior experience from higher education and spending more time on independent self-study has been shown to be associated with study behaviors [8, 9] and academic achievements [5, 6]. Thus, several individual factors appear to be relevant for an understanding of students’ study behaviors and exam grades. However, the evidence concerned with sociodemographic covariates to academic performance across fields of study is inconsistent, as shown in a comprehensive review and meta-analysis covering a range of disciplines and professional courses [2]. Moreover, sociodemographic characteristics such as age and gender do not change like behaviors and environments can. Thus, a stronger focus on contextual factors and what can be done to influence them in ways that support students’ learning, is recommended [10].

Other individual factors associated with academic performance, yet more amenable to change over time, are the students’ approaches to studying. These have been named the deep, strategic, and surface approaches to studying and are well established across a range of settings and academic fields [11–14]. The deep approach is described as studying with the purpose of finding meaning in the topic in question and increasing one’s understanding of it. The strategic approach is oriented towards organized studying, and studying is viewed as instrumental to obtaining high grades. The third approach, the surface approach, describes studying with little reflection, yet with the aim of passing exams while making little true effort [15]. Students often use a combination of attitudes and behaviors related to each of the three approaches; thus, they are not mutually exclusive [16]. Several studies have found study approaches to be significantly associated with academic outcomes. For example, Ward’s studies [17, 18] demonstrated that medical students with who were more inclined to use deep and strategic study approaches obtained better grades, compared to students who were more inclined to use the surface approach. Approaches to studying have also been used to predict occupational therapy students’ exam grades [6]. While five of the study approach subscales were significantly associated with the students’ exam grades, not all associations were as predicted from theory [13, 19]. Moreover, associations between study approaches and academic performance have been found to vary between countries and cultures [20].

The learning environment generally refers to the educational approaches, cultural contexts and physical settings in which teaching and learning takes place. The perceived quality of the learning environment has been shown to have a direct impact on students’ learning and exam results [21–24], as well as students’ satisfaction with the course or study program [23, 25] and students’ personal well-being [26]. However, the evidence for relationships between the learning environment and study performance is mixed. For example, direct associations between learning environment variables and academic grades were non-existing in a Norwegian study of higher education students [1]. On the other hand, studies from various disciplines, including occupational therapy, have consistently demonstrated associations between learning environment factors and students’ approaches to studying [8, 27–31]. For instance, Richardson [32] studied 580 occupational therapy students in seven Danish universities and found that “students’ perceptions of the quality of their courses are in broad terms also positively related to the adoption of desirable approaches and negatively related to the adoption of undesirable approaches” (p. 200). These studies suggest that aspects of the learning environment influence study behaviors and may, in turn, influence academic performance. Moreover, studies have suggested that forms of teaching that modify the learning environment (e.g., group work, problem-based learning, case-study methods) seem to increase deep approach study behaviors [33–35].

In view of the available research evidence, much research effort has been invested in examining associations between study behaviors and measures of academic performance in higher education students. Several studies have also been able to demonstrate associations between the quality of the learning environment and students’ academic performance, but such studies have not yet been conducted with students in occupational therapy. Learning environment factors have been shown to be associated with occupational therapy students’ satisfaction with the education program [25] and their approaches to studying [8, 35], and a possible indirect influence from the learning environment on students’ academic performance has been proposed. However, there is a need to disentangle the potentially interrelated influences of individual and contextual factors on occupational therapy students’ academic performance. By using learning environment factors and approaches to studying as concurrent predictors of occupational therapy students’ academic achievements, this study adds a unique contribution to the health sciences literature.

### Study aim

The aim of the current study was to increase the understanding of the associations between occupational therapy students’ academic performance, and their approaches to studying and perceptions of the learning environment. The research question for the study was: What is the nature of the associations between occupational therapy students’ exam grades, and their approaches to studying, perceptions of the learning environment, and sociodemographic characteristics?

## Methods

### Design and study context

The study is part of a larger and longitudinal study of occupational therapy students’ academic performance, in context of their perceptions of the learning environment and approaches to studying. In the current study, cross-sectional data from students enrolled in the first year of the study program were used.

### Participants and recruitment

First year occupational therapy students at six higher education institutions in Norway were approached for possible inclusion in the study. They were given information about the study in a classroom session and, after providing informed consent to participate, they were asked to complete the questionnaire in session or at a time and place of their own convenience. The data were collected between December 2017 and February 2018.

### Measurement

#### Sociodemographic variables

Age (in years) and time spent on independent studying (average hours during a typical week) were registered as continuous variables. Gender (male/female), having prior experience from higher education (yes/no) and having occupational therapy as the highest prioritized line of education at the time of enrolment (yes/no) were registered as categorical variables.

#### The learning environment

In this study, the Course Experience Questionnaire (CEQ) was used to measure aspects of the learning environment. In its original version, the CEQ consists of 30 items distributed onto five scales: clear goals and standards, emphasis on independence, good teaching, appropriate workload, and appropriate assessment [36]. In addition to the 30 items, one item assesses the students’ general satisfaction with the course. The validated Norwegian translation of the CEQ [37] was used in the present study. The scales indicate that the respondent perceives the course to have (1) clearly established and disseminated goals; (2) high levels of student autonomy and independence; (3) teaching that engages and involves the students; (4) an appropriate workload; and (5) assessment forms that promote and support learning. In view of the preliminary internal consistency results, the ‘appropriate assessment’ scale (Cronbach’s α = 0.45) was removed from the subsequent analyses [38].

#### Approaches to studying

Study approaches were measured with the Approaches and Study Skills Inventory for Students [ASSIST; 19], and the students used a previously validated Norwegian translation of the instrument [39]. The ASSIST consists of 52 statements to which the respondent is asked to rate his or her level of agreement (1 = disagree, 2 = disagree somewhat, 3 = unsure, 4 = agree somewhat, 5 = agree). The instrument has a three-factor structure, recently replicated in a cross-cultural study of undergraduate occupational therapy students [40]. The items are organized accordingly into three main scales (the deep, strategic, and surface approaches to studying). Scale scores are calculated by adding the scores on the relevant items.

#### Exam grades

The students’ average exam grade scores were based on the qualitative descriptors related to the students’ exam grades [41]: fail = 1, sufficient = 2, satisfactory = 3, good = 4, very good = 5, and excellent = 6. As the exam grade measure, we used the students’ grade point average (GPA) by the time the data was collected.

#### Data analysis

The sample was described with means and standard deviations on continuous variables, whereas frequencies and percentages are used on categorical variables. Men and women were compared statistically by Chi-square tests (categorical variables) and independent *t*-tests (continuous variables), and Cohen’s *d* was used as effect size [42]. Generally, *d* ranging 0.20–0.49 are considered small effect sizes, whereas *d* ranging 0.50–0.79 and 0.80 or above are considered medium and large effect sizes, respectively.

To assess the strength of associations between the independent variables and the students’ GPA, a hierarchical linear regression analysis was performed using GPA as outcome variable. In Block 1, representing the background variables, age, gender, educational priority, prior higher education, and time spent on independent study were included as independent variables. In Block 2, representing the perceived learning environment, clear goals and standards, student autonomy, good teaching, and appropriate workload were included. In Block 3, representing the study approaches, the deep approach scale, strategic approach scale, and surface approach scale were included as independent variables. The strength of associations was assessed with the standardized *β* coefficient. The regression models were also used to assess the outcome variance proportions accounted for by each of the models and by all variables together. All analyses were performed with SPSS for Windows, version 26 [43], and results were considered statistically significant if *p* < 0.05.

### Research ethics

The Data Protection Official at the Norwegian Center for Research Data approved the study on October 12, 2017 (project no. 55,875). The participants provided informed consent prior to the commencement of the study and were assured that their information would be treated in confidence.

## Results

### Participants, response rate, and exam grades

A total of 305 students were eligible participants, and of these 187 students (response rate 61.3 %) participated. For each of the institutions, the response rates were 24/76 = 31.6 % in Oslo, 56/77 = 72.7 % in Trondheim, 19/39 = 48.7 % in Gjøvik, 31/47 = 66.0 % in Sandnes, 24/24 = 100.0 % in Tromsø, and 33/45 = 73.3 % in Bergen. Thirteen students had missing values on one or more of the employed variables and were consequently removed, rendering a sample of 174 students for the analysis.

The demographic characteristics and average exam grades of the study participants are displayed in Table 1. The sample was composed predominantly by female students (81 %). The mean age of the participants was 22.7 years (*SD* = 4.3 years), where the male subset had a higher mean age than the female (*p* < 0.01). Compared to the female students, a higher proportion of the male students had prior experience from higher education before enrolment in the occupational therapy program (*p* < 0.05). The female students had obtained significantly better exam grades, compared to the male students (*p* < 0.001).

**Table 1:** Sociodemographic characteristics and average exam grades of the study participants

Variables	All *n* = 174	Men *n* = 33	Women *n* = 141	*p*	*d*
	*M (SD)*	*M (SD)*	*M (SD)*		
Age	22.7 (4.3)	24.6 (5.7)	22.3 (3.8)	< 0.01	0.47
Time spent on self-study	9.1 (6.3)	10.0 (8.1)	8.9 (5.9)	0.38	0.16
Average exam grade	3.9 (1.1)	3.3 (1.2)	4.0 (1.0)	< 0.001	-0.63
	*n* (%)	*n* (%)	*n* (%)		
Had OT as priority education	106 (60.9)	20 (60.6)	86 (61.0)	0.97	
Had prior higher education	73 (42.0)	19 (57.6)	54 (38.3)	< 0.05	

*Note*. *OT* occupational therapy. Time spent on self-study is average number of hours spent during a typical week

### Learning environment and study approaches

In the current study, internal consistency measures (Cronbach’s α) for the learning environment scales were 0.73 (clear goals and standards), 0.63 (emphasis on independence), 0.70 (good teaching), 0.69 (appropriate workload). Cronbach’s α for the study approach scales were 0.71 (deep approach), 0.84 (strategic approach), and 0.76 (surface approach).

The mean ASSIST and CEQ scale scores for the sample are reported in Table 2. There were no significant gender differences related to the learning environment scales. Compared to their male counterparts, the female students showed significantly higher scores on the strategic approach scale (*p* < 0.05).

**Table 2:** The participants’ approaches to studying and perceptions of the learning environment

Variables	All *n* = 174	Men *n* = 33	Women *n* = 141	*p*	*d*
*Study approach scales*	*M (SD)*	*M (SD)*	*M (SD)*		
Deep approach score	56.5 (8.7)	58.5 (11.7)	56.1 (7.8)	0.16	0.24
Strategic approach score	72.2 (10.3)	68.5 (9.2)	73.0 (10.4)	< 0.05	-0.46
Surface approach score	47.5 (9.2)	45.9 (10.0)	47.8 (9.0)	0.28	-0.20
*The learning environment*					
Clear goals and standards	16.6 (3.9)	16.7 (3.9)	16.5 (3.9)	0.84	0.05
Student autonomy	18.6 (4.0)	18.2 (4.4)	18.7 (3.9)	0.58	-0.12
Good teaching	27.3 (6.1)	26.2 (5.3)	27.6 (6.3)	0.27	-0.24
Appropriate workload	15.2 (3.7)	15.4 (3.5)	15.1 (3.7)	0.61	0.08

### Factors associated with exam grades

The results from the regression analysis are displayed in Table 3. Adjusting for all variables in the final model, being female was associated with obtaining better exam results (*β* = 0.22, *p* < 0.01). None of the learning environment scales were associated with the outcome. Among the study approach scales, better exam grades were associated with higher scores on strategic approach (*β* = 0.31, *p* < 0.001), and lower scores on surface approach (*β* = -0.20, *p* < 0.01). The full model explained 27.5 % of the variance in exam grades, with the sociodemographic variables and the study approach scales accounting for similar proportions (11.5 % and 11.2 %, respectively).

**Table 3:** Factors associated with the participants’ grade point average (*n* = 174)

Independent variables	Average exam grade	
*1) Demographics*	Std. *β*	*P*
Age	0.05	0.52
Gender	0.22	< 0.01
Educational priority	0.11	0.11
Prior higher education	0.00	0.99
Time spent on self-study	-0.10	0.20
Explained variance	**11.5 %**	**< 0.01**
*2) The learning environment*		
Clear goals and standards	0.02	0.83
Student autonomy	-0.10	0.25
Good teaching	0.12	0.16
Appropriate workload	-0.03	0.69
R^2^ change	**4.7 %**	**0.06**
Explained variance	**16.3 %**	**< 0.001**
*3) Study approach scales*		
Deep approach score	-0.06	0.44
Strategic approach score	0.31	< 0.001
Surface approach score	-0.20	< 0.01
R^2^ change	**11.2 %**	**< 0.001**
Explained variance	**27.5 %**	**< 0.001**

*Note.* Table content is standardized *β* weights, indicating the strength of each variable’s relationship with average exam grade controlling for all variables in the model, and *p*-values associated with these relationships. Variable coding: male = 0, female = 1; occupational therapy was first priority = 1, occupational therapy was not first priority = 0, prior higher education = 1, no prior higher education = 0. On all other variables, higher scores indicate higher levels

## Discussion

This study investigated how exam grades in a sample of Norwegian occupational therapy students were associated with the students’ background variables, their perceptions of the learning environment and their approaches to studying. None of the learning environment scales were significantly associated with the students’ grades. Adjusting for all variables, we found that better exam grades were associated with being female, and with higher scores on the strategic scale and lower scores on the surface scale.

In view of previous research in other fields and disciplines, we had expected to find associations between some of the learning environment variables and the students’ academic outcomes. For example, in a study using the CEQ, higher perceived workload was previously found to be directly associated with poorer study performance [27]. Another study, in which a different learning environment measure was used, found higher teacher support for student autonomy to be associated with better performance [23]. In our study, despite significant differences in learning environment perceptions being detected across study sites [44], no associations between learning environment factors and study performance were found. These findings imply that variations in academic performance were essentially unrelated to how the students perceived the learning environment. Despite dissimilarities with regards to field of study and measurement methods, these results appear to echo a recent Norwegian study, in which no direct associations between learning environment variables and the students’ academic grades were found [1]. Thus, there is mixed evidence related to the role of the learning environment for students’ academic performance. However, the results of this study should not be interpreted as evidence of the learning environment being irrelevant. In fact, studies have demonstrated associations between learning environment factors and students’ use of study approaches [8, 27–30, 35], suggesting that learning environment factors may influence academic performance indirectly by influencing study behaviors. In addition, their relationship to students’ satisfaction with courses and study programs [23, 25] and students’ well-being [26] strongly suggest that they are important for several good reasons.

The associations between exam grades and the scores on the strategic and surface study approach scales are in line with previous studies in fields such as psychology [12], medicine [17, 18, 44–46], and health science education more in general [48]. The results also mirror the study of associations between study approaches and exam grades in a cross-cultural sample of occupational therapy students [6]. Thus, the study results corroborate the notion that strategic and surface approaches to studying are related to students’ academic performance and supports the notion that these associations are relatively similar across contexts and fields of study. While students with predominant strategic study behaviors tend to perform better academically, those with predominant surface study behaviors perform more poorly.

However, no association was found between the students’ academic performance and their scores on the deep approach scale. While existing theory and research have suggested deep study behaviors and students’ exam grades to be intrinsically linked (e.g., [2, 19, 47, 49]), other researchers have questioned this idea [50, 51]. Moreover, some researchers have expressed a view that the deep-surface dichotomy is overly simplistic, suggesting that study behaviors appear in a broader context (e.g., the nature of the knowledge to be acquired) – thus, the deep approach is not universally preferred [52]. This may have particular relevance for education programs that emphasize practice and skills training, such as the Norwegian occupational therapy programs. Possibly, although extensively used in health and medical education programs across the world [17, 18, 45, 47, 48, 53–56] , the content of the ASSIST scale items may fit better when applied in academic contexts focused on critical thinking concerned with the evidence and rationale for different forms of practice, compared to contexts in which students work on mastering practical skills. For example, a student whose study approach leans largely towards the deep approach in academic contexts may respond differently to items related to reading, linking ideas and comprehensive understanding, while focused on improving practical skills. Although the questionnaires were completed while undergoing campus-based study modules, the study programs’ orientation towards practice and practical skills may have influenced scores on the deep approach scale and how they related to students’ exam grades.

The results of the current study provide support for Ward’s [18] interpretation, suggesting that decreasing surface-type study behaviors among students may be more beneficial for their academic performance than increasing students’ deep-type behaviors. In addition, clusters of study approaches – e.g., having high scores on strategic study behaviors combined with low scores on surface behaviors – may be particularly important in relation to students’ academic performance.

It should be noted that exam grades are not perfect expressions of students’ academic ability. Grades express academic performance within a given assessment context, and according to constructive alignment theory [57], both the frequency and type of assessment are likely to influence the students’ ways of managing the course. Thus, the lack of association between grades and the deep approach scale score may reflect assessment forms used in the occupational therapy programs that do not favor deep learning.

Female students received better exam grades than their male counterparts. Occupational therapy is a profession with a large over-representation of women, among practitioners as well as among students, educators and researchers [4, 58], and it has been suggested that the female dominance in the profession might make it easier for female students to succeed within the established culture [59]. The study points towards a need for conducting more research on the male minority of occupational therapy students, addressing their perceived needs in the study situation and their views on what can assist their success as students and future occupational therapists.

## Study strengths and limitations

A strength of the study is the use of data from all of the occupational therapy education programs in Norway, adding to the generalizability of the results. The total sample was sufficient in size for a multiple regression analysis with a relatively large number of independent variables. However, the response rates varied substantially between education programs, which may have biased the results with more weight given to education institutions with higher response rates. The cross-sectional study design does not allow for causal interpretations of the results. Future studies may examine the extent to which approaches to studying can predict academic outcomes at subsequent assessments, and whether study approaches can mediate relationships between learning environment factors and subsequent academic outcomes. Moreover, research may further explore the situation for male students enrolled in occupational therapy study programs. Finally, given that a substantial outcome variance proportion is left unexplained by the tested regression model, we suggest that future studies include more relevant variables that can account for variance in grades among students. Such variables may include academic achievements prior to those obtained in the current study program, personality factors, and direct measures of study motivation.

## Conclusions

The students’ perceptions of the learning environment were not significantly associated with the grades they obtained, whereas higher scores on the strategic approach scale and lower scores on the surface approach scale were significantly associated with better grades. Occupational therapy students with a desire for obtaining good grades are therefore advised to use strategic study behaviors and avoid using surface approach behaviors. In the development of courses and study programs, educators should strive to organize curricula, assignments and exams in ways that discourage students from relying on a surface approach to studying. They may also routinely assess study behaviors among their students and target students inclined to use the surface study approach for courses or support programs aimed at learning how to study successfully. In addition, organizing student mentoring groups, where successful advanced students share their views and experiences with more inexperienced students, may be useful for students who need support with their studies.

## Data Availability

The datasets generated during and/or analyzed during the current study are not publicly available due to the still ongoing research process but are available from the corresponding author on reasonable request.

## Abbreviations

ASSIST: Approaches and study skills inventory for students
CEQ: Course experience questionnaire
GPA: Grade point average
M: Mean
SD: Standard deviation
